# Differential Proliferation and Maturation of Subcortical Astrocytes During Postnatal Development

**DOI:** 10.3389/fnins.2020.00435

**Published:** 2020-05-08

**Authors:** Temitope Shoneye, Alessandra Tamashiro Orrego, Rachel Jarvis, Yuqin Men, Ming Sum R. Chiang, Yongjie Yang

**Affiliations:** ^1^Department of Neuroscience, Tufts University School of Medicine, Boston, MA, United States; ^2^Graduate School of Biomedical Sciences, Tufts University, Boston, MA, United States

**Keywords:** astrocyte, heterogeneity, development, hypothalamus, proliferation, Sparc

## Abstract

Astrocytes exhibit a region-dependent molecular and functional heterogeneity in the CNS. Although cortical astrocytes proliferate robustly during the first postnatal week and become proliferation quiescent, the temporal proliferation dynamics of astrocytes in subcortical regions during postnatal development remain essentially unknown. Whether subcortical astrocytes mature similarly to cortical astrocytes is also unexplored. In this current study, we examined proliferation of subcortical, especially hypothalamic, astrocytes during postnatal development using genetic labeling of astrocytes and pulse-chase EdU labeling of proliferating cells. While a lower number of proliferating astrocytes was found in the hypothalamus compared to cortex during the first postnatal week, astrocyte proliferation is much more active in hypothalamus than in cortex from P15 to P30 in both proliferating astrocyte density and percentage, indicating a persistent and distinct proliferation pattern of astrocytes in hypothalamus. This observation is further confirmed by Ki67 immunostaining with genetically or immunolabeled astrocytes in hypothalamus and cortex during P15–30. In addition, astrocytes in representative subcortical regions have a modest growth of their domain size and exhibit a significantly smaller domain size compared to cortical astrocytes at P30 when astrocytes have generally completed postnatal maturation. However, the expression of astrocyte-derived Sparc, an important synaptogenic inhibitor, is consistently higher in hypothalamic astrocytes than in cortical astrocytes throughout postnatal development. In summary, our study unveiled a distinct proliferation and maturation pattern of subcortical, especially hypothalamic, astrocytes during postnatal development.

## Introduction

Astrocytes are considered important modulators of brain physiology and pathology, playing diverse and active roles in synaptogenesis, synaptic transmission, and neuronal survival (Clarke and Barres, 2013; Allen and Eroglu, 2017). Interestingly, astrocytes display region-specific differences in their mature morphology and their modulatory functions are also largely associated with local brain regions or circuits (Ben Haim and Rowitch, 2017; Chai et al., 2017; Morel et al., 2017). Several recent studies, by combining RNA-seq with ribosome pull-down (RiboTag or TRAP) (Chai et al., 2017; Morel et al., 2017), cell surface antigen based sorting (John Lin et al., 2017), or single-cell isolation approaches (Zeisel et al., 2018), have systematically characterized transcriptomes of astrocytes from different brain regions. These studies have shown a clear molecular heterogeneity of astrocytes in the adult brain that appears to follow an anatomical dorsal to ventral and anterior to posterior axis (Farmer et al., 2016; Morel et al., 2017). Additionally, astrocytes’ physiological properties, such as gap-junction coupling, inward-rectifying K^+^ currents, and intracellular Ca^2+^ responses are manifested differently across CNS regions (Chai et al., 2017; Oberheim et al., 2012). By using *in vitro* mismatched neuron and astrocyte co-cultures, we further showed that astrocyte-mediated promotion of neurite growth and neuronal synaptic activity is region-conserved (Morel et al., 2017). Whether this molecular, morphological, and functional heterogeneity stems primarily from early stages of astrogliogenesis or is largely influenced by local neighboring signals during the maturation phase remains to be determined.

Subcortical brain regions have a distinct glia to neuron ratio compared with cortex with drastically different neural circuitry (Azevedo et al., 2009). These regions are also highly populated with interneurons derived from medial ganglionic eminence (MGE) progenitors in contrast to predominant glutamatergic neurons in cortex/hippocampus (Bayraktar et al., 2014). Astrocytes are derived from radial glia (RG) in the CNS when RGs transition from GLAST^+^/Nestin^+^ to GLAST^+^/Nestin^–^ progenitors during late embryonic or early postnatal stages (Bayraktar et al., 2014; Siddiqi et al., 2014). Although astrocytes proliferate robustly during the first 2 weeks postnatally to occupy the cortex, presumably through a local proliferation mechanism from newly born immature astrocytes (Ge et al., 2012), as RGs are heterogenous with a dorsoventral (DV) distribution along the ventricular zone (VZ), whether subcortical astrocytes undergo similar proliferation dynamics during early postnatal development has not been explored. In addition, newly born cortical astrocytes undergo a maturation phase to acquire their uniquely ramified morphology and express important functional proteins such as excitatory amino acid transporter (EAAT2) (Morel et al., 2014). Whether these morphological and molecular changes similarly occur for subcortical astrocytes remains to be investigated.

In the current study, we performed genetic and 5-ethynyl-2′-deoxyuridine (EdU) pulse-chase labeling to investigate astroglial proliferation dynamics in developing subcortical regions. We also examined postnatal morphological and molecular changes of subcortical astrocytes.

## Materials and Methods

### Animals

The Ai14-tdT^f/f^ reporter, Bac *Slc1a3*-CreERT transgenic (C57BL/6) and Bac *Aldh1l1*-eGFP mice were obtained from the Jackson Laboratory. VGluT1^–/–^ mice were obtained as a kind gift of Dr. Robert Edwards (University of California, San Francisco). The EAAT2-tdTomato (tdT) transgenic mice were generated as previously described (Yang et al., 2011). Animals were deeply anesthetized with ketamine (100 mg/kg) plus xylazine (10 mg/kg) in saline by intraperitoneal injection and perfused intracardially with 4% PFA in PBS. The brains were dissected and kept in 4% PFA overnight at 4°C, then cryoprotected by immersion in 30% sucrose for 48 h. Brains were embedded and frozen in OCT-Compound Tissue-Tek (Sakura). Sagittal sections (20 μm) were prepared with a cryostat (Leica HM525) and mounted on glass SuperFrost^+^ slides (Thermo Fisher Scientific). Mice of both sexes were used for all experiments. All procedures were in strict accordance with the National Institutes of Health *Guide for the Care and Use of Laboratory Animals* and were approved by the Tufts University Institutional Animal Care and Use Committee.

### Tamoxifen and EdU Injection

Tamoxifen (4-OHT; Sigma-Aldrich) was suspended at 20 mg/ml in ethanol and diluted into sunflower seed oil at a final concentration of 2 mg/ml in 10% ethanol. For *Slc1a3*-CreERT^+^Ai14^f/+^ mice, an intraperitoneal injection of 10 μl 4-OHT (50 mg/kg) was administered from P1 to P2 for a total dose of 0.25 mg to selectively label astrocytes. The Click-iT EdU Alexa Fluor 488 Imaging Kit was suspended at 2.5 mg/ml in DMSO, a 1:10 dilution from the stock, and the final concentration was 10 mg/kg. An intraperitoneal injection of EdU was administered at different developmental time points (P3, P8, and P15) for a total dose of 0.25 mg.

### Immunohistochemistry

Mice were perfused by intracardial perfusion with 4% paraformaldehyde in 1× PBS. Brains were cut into 20 μm sections with a cryostat. Slides were rinsed three times in PBS for 10 min each, then incubated with blocking buffer (1% BSA, 5% normal goat or donkey serum, and 0.2% Triton X-100 in PBS) for 1 h at room temperature (RT). Primary antibodies against Ki-67 (1:100 rabbit anti-Ki-67, Pierce #PA5-19462), Sparc (1:500 goat anti-Sparc, R&D Systems #AF942), or Sox9 (1:100 goat anti-Sox9, R&D Systems #AF3075) were incubated overnight at 4°C in the appropriate blocking buffer. After washing slides three times in PBS, secondary antibody (1:2000, donkey anti-goat Alexa Fluor 647 or goat anti-rabbit Alexa Fluor 488, Life Technology) was added for 2 h at RT. For EdU immunostaining, slides were permeabilized with 0.5% Triton X-100 in PBS for 20 min at RT. The slides were then washed with 3% BSA in PBS twice for 5 min each. The reaction cocktail was added for 30 min at RT. The sections were rinsed once in BSA for 5 min before mounting.

### Acquisition of Images and Quantification of Labeled Cells

Images were obtained with a confocal laser scanning microscope (A1R, Nikon), Keyence BZ-X700 microscope, or Zeiss AXIO Imager with ApoTome. For EdU quantification, Keyence stitched images of the entire sagittal sections were taken with a 10× objective lens and all cells were manually counted using Fiji ImageJ (multi point tool). We counted all tdT^+^ cells (astrocytes), EDU^+^ cells (dividing cells), and tdT^+^EDU^+^ cells (dividing astrocytes) in cortex, thalamus, and hypothalamus. At each time point we calculated the density of astrocytes, dividing cells, and dividing astrocytes by dividing the number of cells by the area of the respective brain region to determine the number of cells per mm^2^. We also quantified the percentage of dividing astrocytes among all labeled astrocytes at P30 by dividing the number of tdT^+^EdU^+^ cells by the total number of tdT^+^ cells. For Ki-67 quantification, Keyence stitched images of sagittal brain sections were taken with a 10× objective, and a 0.5 mm^2^ grid was overlaid on the image. tdT^+^ cells (astrocytes) and tdT^+^Ki-67^+^ cells (dividing astrocytes) were quantified from 20 grids in cortex and 10 grids in hypothalamus, and percentage of dividing astrocytes among labeled astrocytes was determined. Labeling efficiency of the Ai14-tdT reporter in a given brain region was estimated by dividing density of tdT^+^ cells by the density of eGFP^+^ cells in age-matched Bac *Aldh1l1*-eGFP mice.

For Sparc analysis, images were taken using the Zeiss microscope with a 20× objective. The numbers of eGFP^+^SPARC^+^ cells were individually quantified in Fiji ImageJ and the intensity of Sparc from co-localized astrocytes (indicated with eGFP fluorescence) was also measured. Sparc fluorescence-negative area in each image was selected to determine the background and subtracted. In Fiji, maximum projections were generated from Zeiss images for each channel and the merged images. The number of eGFP^+^ labeled astrocytes in the field was manually counted using the region of interest (ROI) manager. The freehand selection tool was then used to outline labeling in Sparc images that were co-localized with eGFP^+^ labeled astrocytes in the merged images. Measurements of average intensity within the region were calculated through Fiji’s ROI manager using the polygon selection tools to draw a circle around the cells.

### Astrocyte Domain Analysis

All confocal images for Imaris analysis were taken with a 40×oil-immersion objective lens. Images were taken under optimized setting to best show the astrocyte morphology. The settings were consistent across all age groups. For the morphological analysis of astrocytes, a 3D reconstruction was first generated using the original confocal *Z*-stack images in Imaris software. The surface tool was then used to build the domain. This function uses an automatic smoothing of the image with the Gaussian filter. A tdT fluorescence-negative area in each of the confocal stack images was used as the internal control to determine the background fluorescence. The sensitivity threshold (absolute intensity) was manually adjusted so that the generated astrocyte domains in the 3D images matched with the original confocal images. The cell somas were then detected based on size (≤12 μm in diameter) and used as seeding points to build the 3D domain. The quality (intensity) threshold was also manually adjusted to ensure that all cell somas were detected in a given image. The seeded watershed algorithm enables the Imaris software to recognize and split the domains of neighboring cells. Cells that were only partially included in confocal and 3D images were excluded from analysis. The volume size of individual astrocytes can be directly measured from generated 3D domains in Imaris.

### Statistical Analysis

Sample size and statistical approach used for each experiment are described in figure legends. All analysis was performed using GraphPad Prism 7. All values were plotted as mean ± SEM, except for the astrocyte domain size values, which were converted to cumulative frequency. The Kolmogorov–Smirnov test was used to analyze significance for all cumulative frequency curves. For multiple groups (>2), one-way ANOVA was used to analyze the variance, followed by a Tukey *post hoc* test to compare multiple groups. For two-group comparison, an unpaired two-tailed *t*-test was used. Statistical significance was tested at a 95% (*p* < 0.05) confidence level and the exact *p*-values are presented in each figure panel and legend.

## Results

### Proliferation of Subcortical Astrocytes During Early Postnatal Development

**FIGURE 1:**
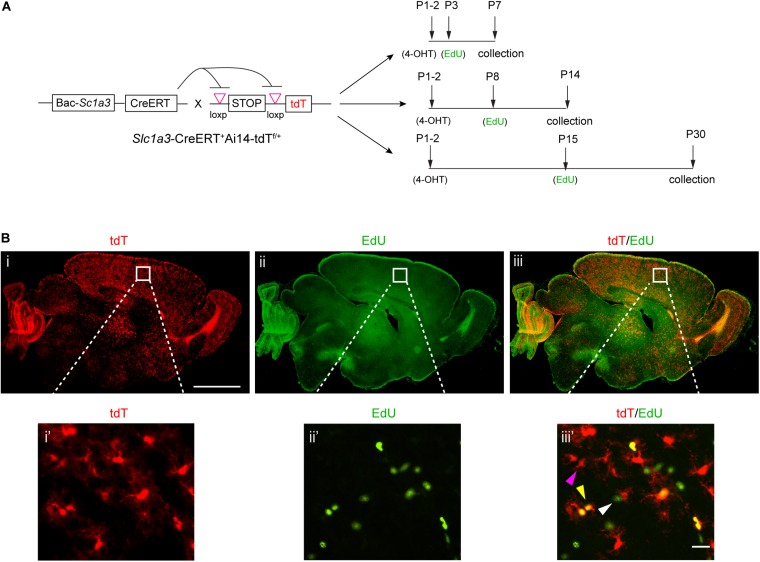
Genetic labeling of astrocytes and EdU pulse-chase labeling of proliferating cells during postnatal development. **(A)** Experimental paradigm for genetic labeling of astrocytes and EdU pulse-chase labeling of proliferating cells at different time points postnatally; specific time points for 4-OHT and EdU injections are indicated. **(B)** Representative images of astrocyte labeling with the tdT reporter in *slc1a3*-CreERT^+^Ai14-tdT^f/+^ mice and EdU labeling of proliferating cells in P7 brain; magnified images show cortical astrocytes. Magenta arrow, tdT^+^EdU^–^ cell; white arrow, tdT^–^EdU^+^ cell; yellow arrow, tdT^+^EdU^+^ cell. Scale bar: 1 mm (i–iii), 10 μm (i’–iii’).

Previous studies found that the peak of astrocyte proliferation in the cortex occurs within the first postnatal week, after which these astrocytes become gradually proliferation quiescent (Ge et al., 2012). To examine the proliferation dynamics of subcortical astrocytes during postnatal development, we combined the genetic labeling of astrocytes using Cre-dependent Ai14-tdT^f/f^ mice and EdU pulse-chase for labeling proliferating cells (Figure 1A). Although astrocytes can be conventionally identified by immunostaining of glial fibrillary acidic protein (GFAP) and recently aldehyde dehydrogenase L1 (ALDH1L1) (Cahoy et al., 2008; Yang et al., 2011), these immunostaining signals can be incomplete (in the case of GFAP) (Fujita et al., 2014) or weak (in the case of ALDH1L1) (Yang et al., 2011) and often more evident in astroglial processes than the cell body, making it ambiguous to clearly identify and quantify individual astrocytes. Alternatively, we bred Bac *Slc1a3* CreERT transgenic mice with Ai14-tdTomato (tdT) reporter mice in which the tdT reporter can be induced in a Cre-dependent manner in astroglial soma and processes, facilitating the confident quantification of individual astrocytes in the CNS. Glutamate transporters GLAST and GLT1 (human analog EAAT1 and EAAT2, encoded by *Slc1a3* and *Slc1a2*, respectively) are both highly and selectively expressed in astrocytes during postnatal development in the CNS (Rothstein et al., 1994). Although the *Slc1a3* genomic promoter is also active in RG during late embryogenesis (Regan et al., 2007), RG’s fate is destined toward astrocytes at P1–2 (Rowitch and Kriegstein, 2010) when 4-hydroxy-tamoxifen (4-OHT) was administered. Consequently, it is unlikely that *Slc1a3*-Cre induced tdT is expressed in other CNS cell types in *Slc1a3*-CreERT^+^Ai14-tdT^f/+^ mice. We have also previously performed immunostaining with cell-type specific markers to confirm that tdT is indeed expressed in astrocytes, but not in other CNS cells, in cortex (Higashimori et al., 2016). In addition to astrocyte labeling, we also performed a single intraperitoneal (i.p.) injection of EdU to *Slc1a3*-CreERT^+^Ai14-tdT^f/+^ mice. EdU is a nucleotide analog that can be incorporated into DNA during the DNA synthesis phase of the cell cycle, thus reliably and selectively labeling proliferating cells. Although EdU-mediated labeling of proliferating cells can continue for more than one cycle of division, its quantification still allows us to assess the overall proliferation activity in a given time period.

To examine astrocyte proliferation within a specified postnatal period of time, we decided to inject EdU at P3, P8, or P15 to label proliferating cells during the postnatal period from P3 to P7 (week 1), P8 to P14 (week 2), and P15 to P30 (weeks 3–4), respectively (Figure 1A). Based on the previously observed significant decrease of cortical astrocyte proliferation from P15 to P30, we decide to combine weeks 3 and 4 in assessing astrocyte proliferation activity during this time period. In all experimental groups, 4-OHT was injected at P1–2 to selectively induce expression of tdT in astrocytes. With the combined injections of 4-OHT and EdU, proliferating astrocytes, -likely from multiple cycles of divisions, are labeled as tdT^+^EdU^+^ cells that can be unbiasedly quantified to reflect the proliferation activity of astrocytes during indicated periods (Figure 1A) of early postnatal development. We tested combinations of dose and frequency of EdU and 4-OHT injections to achieve optimal numbers of cell labeling for quantification. The combined injections of 4-OHT and EdU resulted in efficient induction of tdT expression in astrocytes (Figure 1Bi and the magnified view i’) and sufficient labeling of proliferating cells (Figure 1Bii and the magnified view ii’) in the CNS. Co-localized tdT and EdU labeled (tdT^+^EdU^+^, yellow arrow), tdT^+^ alone (tdT^+^EdU^–^, magenta arrow), or EdU^+^ alone (tdT^–^EdU^+^, white arrow) cells were all observed (Figure 1Biii’). Although our strategy is not designed to label all astrocytes or proliferating cells, the number of labeled astrocytes and proliferating cells is sufficient for examining astrocyte proliferation activity in both cortex and subcortical regions. On the other hand, it is known that other CNS cell types, particularly polydendrocyte NG2 cells, also actively proliferate during early postnatal development (Kang et al., 2010), thus it is not unexpected that some EdU^+^ proliferating cells do not overlap with tdT^+^ astrocytes. In addition, it is also possible that not all tdT^+^ astrocytes were sampled by the EdU injection or underwent division at the time when EdU was injected. Overall, the combined labeling of astrocytes and proliferating cells allows temporal and spatial quantification of proliferating astrocytes, as an indication of overall astrocyte proliferation activity, during early postnatal development.

**FIGURE 2:**
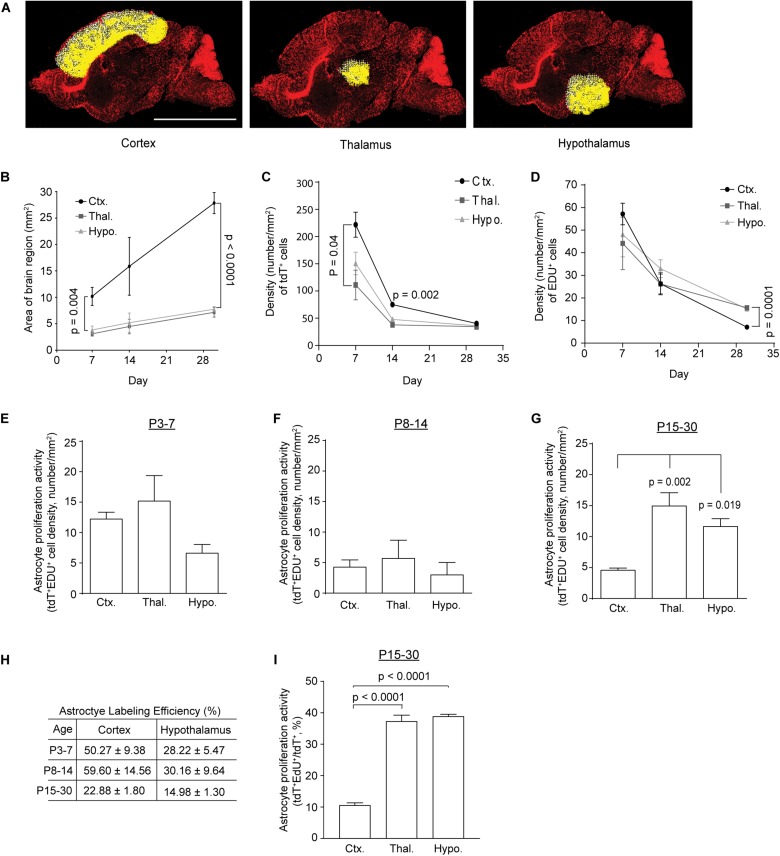
Temporal proliferation dynamics of subcortical astrocytes during postnatal development. **(A)** Representative images highlighting the cortex, thalamus, and hypothalamus for quantifying proliferating cells; scale bar: 2 mm. Each white cross with a yellow dot represents an individual cell. **(B)** Changes in the size of cortex, thalamus, and hypothalamus during early postnatal development. One-way ANOVA with Tukey’s *post hoc* test; significant differences between the means at P7 (*p* = 0.001, *F*_(2,12)_ = 12.49) and P30 (*p* < 0.0001, *F*_(2,9)_ = 482.38). Density of tdT^+^ [significant differences between the means at P7 (*p* = 0.044, *F*_(2,7)_ = 5.027) and P14 (*p* = 0.002, *F*_(2,4)_ = 49.8)] **(C)** or EDU^+^ [significant difference between the means at P30 (*p* < 0.0001, *F*_(2,9)_ = 34.85)] **(D)** cells in cortex, thalamus, and hypothalamus during postnatal development. *p*-values determined by one-way ANOVA and *post hoc* Tukey’s test. Density of tdT^+^EdU^+^ cells in cortex, thalamus, or hypothalamus generated from P3–7 **(E)**, P8–14 **(F)**, and P15–30 **(G)**; one-way ANOVA followed by *post hoc* Tukey’s test, significant difference between the means at P30 (*p* = 0.002, *F*_(2,9)_ = 13.2). **(H)** Calculated genetic labeling efficiency of astrocytes from P3–7, P8–14, and P15–30 in cortex and hypothalamus. **(I)** Percentage of proliferating astrocytes (tdT^+^EdU^+^/tdT^+^) in cortex, thalamus, or hypothalamus from P15–30; one-way ANOVA with Tukey’s *post hoc* test; significant differences between the means (*p* < 0.0001, *F*_(2,8)_ = 140.8). *n* = 3 images/mouse for 4–5 individual mice. A total of 3000–5000 tdT^+^ or EDU^+^ cells were quantified from three images/mouse per region/time point (a total of 4–5 mice per condition). All *p*-values shown in the figure were determined by *post hoc* analysis, and if not otherwise specified represent a comparison between cortex and thalamus.

To analyze astrocyte proliferation in subcortical regions, especially thalamus and hypothalamus, we prepared sagittal sections from *Slc1a3*-CreERT^+^Ai14-tdT^f/+^ mice following 4-OHT and EdU injections at different time points. The total number of tdT^+^, EdU^+^, and tT^+^EdU^+^ cells were quantified from the whole thalamus, hypothalamus, and cortex regions, highlighted with white crosses and yellow dots (indicating individual cells, Figure 2A) using the ROI script in ImageJ. As the brain regions analyzed undergo rapid expansion during early postnatal development, we first measured the size of the quantified brain regions and found that the cortex region expands substantially faster (slope of linear regression = 0.76 mm^2^/day, Figure 2B) than subcortical regions (slope of linear regression = 0.17 or 0.19 mm^2^/day for hypothalamus or thalamus, respectively, Figure 2B). The area of these brain regions was also used to calculate the density of total tdT^+^, EdU^+^, and tdT^+^EdU^+^ cells in these regions. We found that the density (number of cells/mm^2^) of tdT^+^ astrocytes was highest in cortex compared to thalamus and hypothalamus at P7 and P14 (Figure 2C). The density of astrocytes significantly decreased in all regions examined as brain volume rapidly increases in postnatal weeks 2–4 (Figure 2C). Although the density of EdU^+^ cells was generally comparable in all regions examined at P7 and P14 (Figure 2D), it was significantly higher in thalamus and hypothalamus than in cortex at P30 (Figure 2D), suggesting that proliferation activity in these regions remains active while cortical proliferation is much reduced from P15 to P30. It is noted that the density of EdU^+^ cells at P7 is highest in the cortex, though not significantly different from that in thalamus and hypothalamus, possibly due to the incomplete labeling of all proliferating cells. Similarly, although the density of tdT^+^EdU^+^ cells, presumably proliferating astrocytes, is higher in cortex than that in hypothalamus at P7 (Figure 2E), the density of tdT^+^EdU^+^ cells becomes significantly higher in thalamus (*p* = 0.002) and hypothalamus (*p* = 0.019) than in cortex at P30 (Figure 2G). The density of tdT^+^EdU^+^ astrocytes is comparable across all examined regions at P14 (Figure 2F).

Although the *Slc1a3* promoter is widely active in astrocytes in the CNS, recent studies have indicated a dorsal to ventral axis heterogeneity in astrocyte gene expression (Farmer et al., 2016; Morel et al., 2017) in which the *Slc1a3* promoter could be heterogeneously activated in cortical and subcortical astrocytes. As a result, this potential *Slc1a3* promoter activity heterogeneity may become a confounding factor in quantifying genetically labeled astrocytes. We then decided to determine whether there is a comparable genetic labeling efficiency of astrocytes in cortex and hypothalamus in *Slc1a3*-CreER^+^Ai14-tdT^f/+^ mice. As it is likely that tdT^+^-mediated labeling of astrocytes is incomplete with *Slc1a3*-CreER^+^Ai14-tdT^+^ tamoxifen-injected mice, we employed Bac *Aldh1l1*-eGFP astrocyte reporter mice in which most if not all astrocytes are labeled with eGFP across the CNS based on previous studies (Cahoy et al., 2008), and quantified the number of eGFP^+^ astrocytes in cortex and hypothalamus respectively in a size-matched area as in *Slc1a3*-CreER^+^Ai14-tdT^+^ mice. Interestingly, although cortex size expands significantly faster than hypothalamus during postnatal development (P7–30, Figure 2B), our quantification consistently found that cortical astrocyte density (# of astrocytes/mm^2^) is only 74–80% of hypothalamic astrocyte density during the same time period depending on the exact time point examined. Based on eGFP^+^ and tdT^+^ astrocyte numbers from Bac *Aldh1l1*-eGFP and *Slc1a3*-CreER^+^Ai-14-tdT^f/+^ mice in corresponding regions (size-matched) and time points, we calculated the genetic labeling efficiency of astrocytes in cortex and hypothalamus from P3 to P7, P8 to P14, and P15 to P30 (Figure 2H) and found that the genetic labeling efficiency of astrocytes in cortex is typically 1.5 to 2-fold higher than that of hypothalamic astrocytes (Figure 2H). To eliminate the influence of the differential genetic labeling efficiency of astrocytes in these regions on the analysis, we calculated the percentage of proliferating astrocytes (tdT^+^EdU^+^/tdT^+^) and found that both hypothalamus and thalamus have a substantially higher percentage of proliferating astrocytes (*p* < 0.0001) than in cortex from P15 to P30 (Figure 2I), consistent with the density-based proliferative astrocyte analysis in Figure 2G.

**FIGURE 3:**
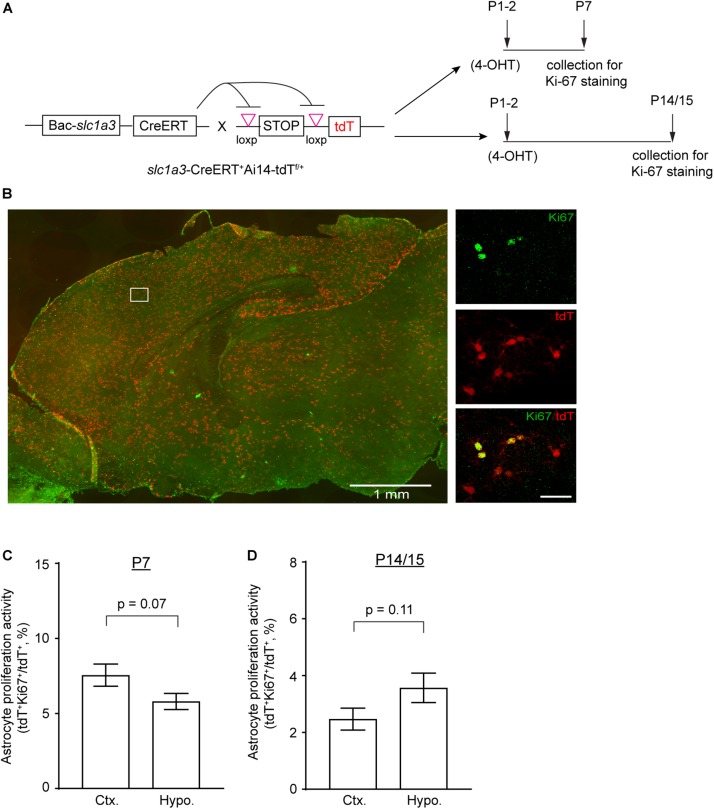
Astrocyte proliferation activity in cortex and hypothalamus determined by genetic labeling of astrocytes and Ki67 immunostaining. **(A)** Experimental strategy for genetic labeling of astrocytes and Ki-67 staining of proliferating cells at different postnatal time points. **(B)** Representative images of astrocyte labeling with the tdT reporter in *Slc1a3*-CreERT^+^Ai14-tdT^f/+^ mice and Ki-67 labeling of actively proliferating cells in P7 brain; magnified images show cortical astrocytes. Scale bars: 1 mm (left panel), 30 μm (magnified images). Percentage of proliferating astrocytes (100 × tdT^+^Ki-67^+^/tdT^+^) in cortex and hypothalamus at P7 **(C)** and P14/P15 **(D)**; *n* = 3 images/mouse from 3–4 individual mice per region/time point. *p*-values determined by unpaired two-tailed *t*-test.

As it is likely that EdU-mediated labeling could pass several cycles of cell division, to further assess astrocyte proliferation activity in cortex and hypothalamus during early postnatal development, we performed immunostaining of Ki67, a nuclear marker of active proliferation, on cortical and hypothalamic sections of *Slc1a3*-CreER^+^Ai14-tdT^+^ mice at different time points of postnatal development (Figure 3A) to provide a snapshot assessment of the proliferating astrocytes in these regions. As shown in Figure 3B, we observed widespread labeling of tdT^+^ astrocytes in brain and clear co-localization of tdT reporter with Ki67 immunoreactivity, an indication of proliferative astrocytes. The quantification of total tdT^+^ and tdT^+^Ki67^+^ astrocytes showed 25% more proliferating astrocytes in cortex than in hypothalamus at P7 (Figure 3C), while 40% more proliferating astrocytes were observed in hypothalamus than in cortex at P14/P15 (Figure 3D). To assess whether astrocytes at cortex and hypothalamus still actively divide at P30 and beyond, we further performed immunostaining of Ki67 and Sox9, a recently characterized nuclear marker of adult astrocytes (Sun et al., 2017), and found essentially no Ki67^+^ cells or Ki67^+^Sox9^+^ astrocytes at P30 in both cortex and hypothalamus (data not shown), suggesting that astrocytes in these regions become essentially proliferatively quiescent at P30 and beyond. These Ki67 snapshot analysis results further support our EdU-based analysis that more cortical astrocytes are proliferatively active than hypothalamic astrocytes at P7, but hypothalamic astrocytes are significantly more active in proliferation than cortical astrocytes from P15 to P30. Taken together, these results unveil distinct dynamics of astrocyte proliferation in subcortical regions of thalamus and particularly hypothalamus in comparison to cortical astrocytes. Consistent with previous observations (Ge et al., 2012), we found that cortical astrocytes robustly proliferate early at P7, but that the proliferation activity of these astrocytes is drastically reduced after the first week. In contrast, astrocytes in thalamus and particularly hypothalamus become more active in proliferation than cortical astrocytes later in early postnatal development.

### Morphological Maturation of Subcortical Astrocytes During Postnatal Development

**FIGURE 4:**
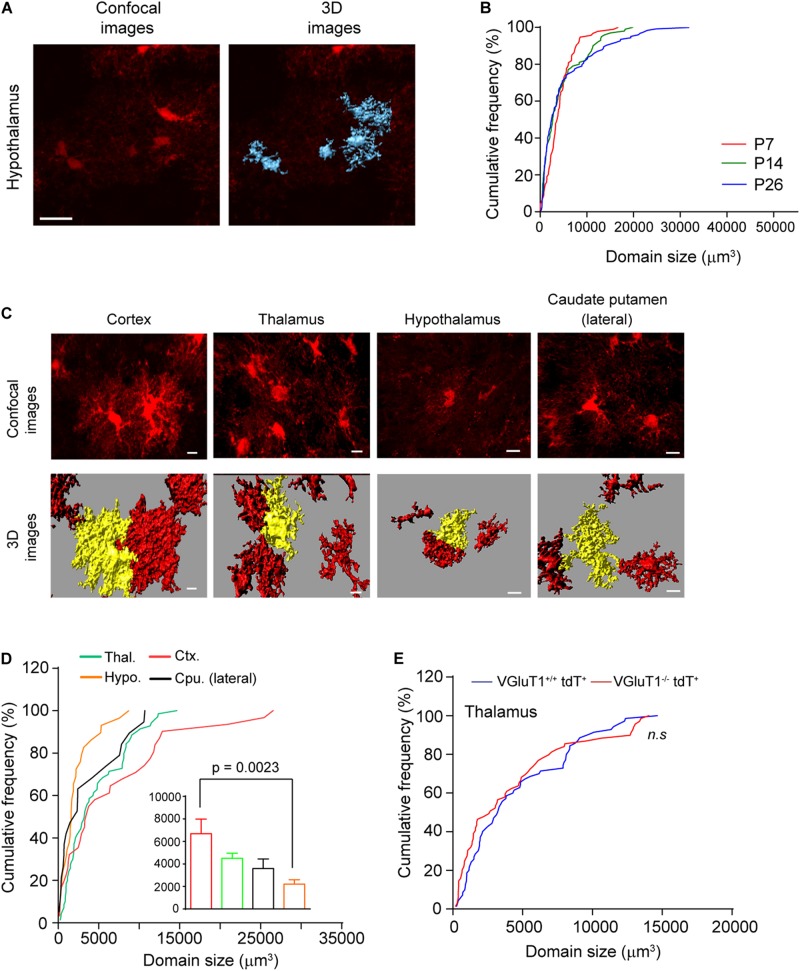
Morphological maturation of subcortical astrocytes during postnatal development. **(A)** Representative confocal and 3D images of astrocytes from hypothalamus of *eaat2*-tdT mice. Scale bar: 20 μm; **(B)** cumulative frequency curve of astroglial domain size in hypothalamus at different postnatal developmental time points (P7, P14, and P26). *n* = 60 astrocytes/group from multiple mice; **(C)** representative confocal and 3D images of astrocytes from different brain regions of *eaat2*-tdT mice at P30. Scale bar: 20 μm; **(D)** cumulative frequency curve of astroglial domain size in these brain regions. The insert bar graph represents the average astroglial domain size (one-way ANOVA, significant differences between the means, *p* = 0.002, *F*_(3,145)_ = 5.220). *n* = 50–84 astroglia/group from multiple mice; *p*-value in the figure determined by *post hoc* Tukey’s test. **(E)** Cumulative frequency curve of thalamic astroglial domain size in VGluT1^–/–^tdT^+^ and VGluT1^+/+^tdT^+^ mice. *n* = 70 astrocytes/group from multiple mice; n.s., not significant.

Previous studies have found that cortical and hippocampal astrocytes undergo a maturation process during which the cell domain size significantly increases by growing abundant fine processes (Bushong et al., 2002; Morel et al., 2014). To determine whether subcortical astrocytes also undergo a similar morphological maturation process, we quantified hypothalamic astrocyte domain size from confocal images of EAAT2-tdT astrocyte reporter mice. We have previously shown that the tdT reporter is able to illustrate the full morphology of mature astrocytes, allowing direct measurement of the domain size (volume) of individual astrocytes from confocal images using Imaris image analysis software (Morel et al., 2014). Representative confocal and 3D Imaris images from hypothalamic astrocytes of EAAT2-tdT mice are shown in Figure 4A. Subsequent quantification of domain size found that a majority of hypothalamic astrocytes have similar domain size at P7, P14, and P26, with only a small portion of astrocytes (∼20%) substantially growing their domain size from P7 to P26 (Figure 4B). These results reveal for the first time that hypothalamic astrocyte domain size only has a very modest growth during postnatal development, in sharp contrast to the dramatic growth of cortical astrocyte domain size during the same developmental period.

In addition to the small hypothalamic astrocyte domain size at P26, to gain insights about the morphological features of astrocytes in other subcortical regions, we next measured the domain size of astrocytes from other representative subcortical regions (thalamus and lateral caudate putamen) from EAAT2-tdT mice, as shown in Figure 4C. The astrocytes in these subcortical regions appear morphologically less complex than those in cortex at P30 (Figure 4C). In particular, the enormous arborization of astroglial branches typically found in cortical astrocytes was not evident in astrocytes from other regions (Figure 4C). Subsequent quantification confirmed that astroglial domain size is smallest in the hypothalamus (2212 mm^3^) and largest in the cortex (6687 mm^3^) (Figure 4D). We previously showed that the arborization of cortical astroglial branches can be regulated by local VGluT1^+^ neuronal glutamatergic signaling (Morel et al., 2014). To determine whether astrocytes in subcortical regions are regulated by the same mechanism, we quantified astroglial domain size in the thalamus from VGluT1^+/+^tdT^+^ and VGluT1^–/–^tdT^+^ mice at P30. We and others have previously shown that the loss of VGluT1 drastically reduces glutamatergic signaling in the CNS (Fremeau et al., 2004). As shown in Figure 4E, the distribution of thalamic astroglial domain size is similar in VGluT1^–/–^tdT^+^ and VGluT1^+/+^tdT^+^ mice, suggesting that thalamic astroglia domain size is not influenced by the loss of neuronal VGluT1^+^ glutamatergic signaling. We previously also observed similar results in hypothalamic astrocytes of VGluT1^–/–^tdT^+^ and VGluT1^+/+^tdT^+^ mice (Morel et al., 2014). The selective effect of VGluT1^+^ neuronal signaling on cortical but not on thalamic (and hypothalamic) astrocyte domain size indicates a region-specific regulatory mechanism for astrocyte morphological maturation during postnatal development.

**FIGURE 5:**
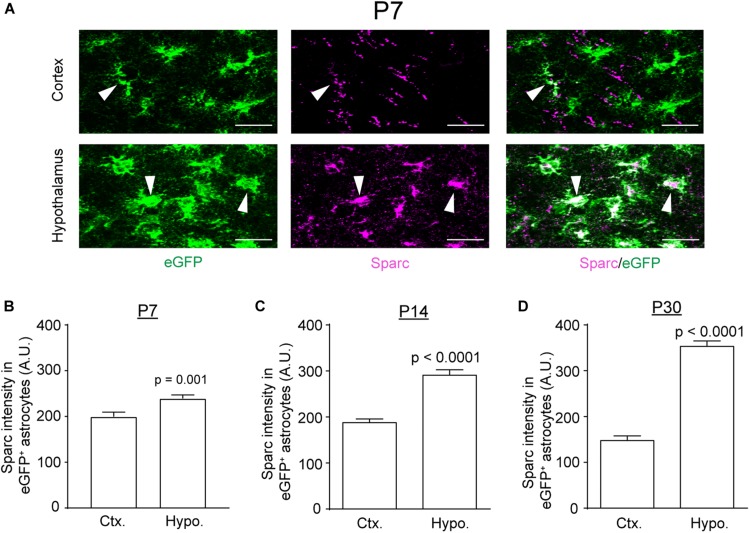
Differential expression of Sparc in cortical and hypothalamic astrocytes during postnatal development. **(A)** Representative images of Sparc immunostaining in cortex and hypothalamus of Bac *Aldh1l1*-eGFP astrocyte reporter mice at P7; white arrows, positive Sparc immunostaining in eGFP^+^ astrocytes; scale bar: 50 μm. Quantification of Sparc intensity in eGFP^+^ astrocytes in cortex and hypothalamus at P7 **(B)**, P14 **(C)**, and P30 **(D)**. *n* = 118–134 astrocytes/group from multiple mice; *p*-values were determined by unpaired two-tailed *t*-test.

We recently profiled translating mRNAs in adult astrocytes from multiple brain regions, through which we identified several genes that are differentially expressed in astrocytes across brain regions (Morel et al., 2017). In particular, we found that the expression of Sparc, one of astrocyte-secreted modulators of synaptogenesis (Allen and Eroglu, 2017), is substantially higher in adult subcortical (hypothalamic and thalamic) astrocytes than in cortical and hippocampal astrocytes. To determine whether the differential expression pattern of Sparc in these regions stems from earlier developmental stages, we performed immunostaining of Sparc in cortex and hypothalamus of Bac *Aldh1l1*-eGFP astroglial reporter mice at different developmental time points (P7, P14, and P30). As shown in Figure 5A, Sparc immunoreactivity is widely co-localized with eGFP^+^ astrocytes (white arrows) in both regions at P7 (representative images for other developmental time points not shown). Quantification of Sparc immunoreactivity in eGFP^+^ astrocytes showed that Sparc immunoreactivity in hypothalamic astrocytes is significantly higher than that in cortical astrocytes as early as P7 and persists at P14 and P30 (Figure 5B–D), suggesting that the differential expression pattern of Sparc in astrocytes starts in early astrogliogenesis and continues through postnatal development into the adult.

## Discussion

In the current study, we investigated the postnatal proliferation and maturation of astrocytes in subcortical regions by employing astrocyte genetic and EdU pulse-chase labeling. In contrast to the robust proliferation of cortical astrocytes within the first postnatal week, we found that astrocytes in subcortical regions, particularly in hypothalamus are less proliferatively active than cortical astrocytes during the same period. However, a significantly higher percentage of hypothalamic astrocytes remain proliferatively active from P15 to P30 than cortical astrocytes, indicating that hypothalamic astrocytes have a distinct temporal, particularly a more persistent proliferation dynamic in comparison to cortical astrocytes. This is also in parallel to our quantification that hypothalamic astrocyte density is 20–26% higher than cortical astrocyte density throughout postnatal development. As the area of cortex expands at a faster rate than subcortical regions (Figure 2B), it is unlikely that the increased percentage of proliferative astrocytes in subcortical regions from P15 to P30 is due to a greater territorial expansion of those brain regions compared to cortex.

Although our combined use of astrocyte genetic and EdU pulse-chase labeling effectively labels proliferating astrocytes, as we intended to assess the proliferation activity of astrocytes but not to label all proliferating cells at a given time period, we decide not to inject a high dose of EdU or perform repeated injections to label all proliferating cells. Similarly, our EdU injection dose also serves to sample but not label all proliferating cells. As a result, it is possible that not all dividing tdT^+^ astrocytes were sampled by the EdU injection or underwent division at the time when EdU was injected. In addition, as EdU likely labels more than one cycle of dividing cells, EdU-based quantification may include the number of proliferating astrocytes from multiple generations, which is different from the single-day snapshot quantification based on Ki67 immunostaining. Despite the difference in quantifying proliferative astrocytes, both approaches showed similar results that astrocyte proliferation activity is switched in cortex and hypothalamus during early postnatal development. Given the observation that there is a higher percentage of proliferative astrocytes at P14/P15 in hypothalamus and that there are essentially no proliferative astrocytes at P30 in both regions (based on Ki67/Sox9 staining and (Ge et al., 2012), it is likely that hypothalamic astrocytes are persistently more active than cortical astrocytes from P15 until astrocytes from both regions become quiescent near the P30 time point.

The distinct proliferation dynamics between cortical and subcortical (hypothalamic) astrocytes are likely to closely associate with the unique characteristics of synaptogenesis in each brain region. Immature (but not mature) cortical astrocytes are known to secret extracellular matrix proteins, such as thrombospondin (Thbs), hevin, and glypican, etc., (Allen and Eroglu, 2017) to actively promote the formation and function of glutamatergic synaptogenesis that is the dominant synapse type in cortex. Therefore, the massive generation of immature cortical astrocytes in a relatively short time period may potentially facilitate the supply of such extracellular proteins to promote glutamatergic synaptogenesis in the cortex. In contrast, as interneurons are more widely distributed in hypothalamus (Obrietan and van den Pol, 1995) and Thbs/hevin/glypican have no apparent effect on promoting GABAergic synaptogenesis (Allen and Eroglu, 2017), it is not unexpected that hypothalamic astrocyte proliferative activity is low at first. In addition to the distinct proliferation dynamics, we found that Sparc expression is significantly higher in hypothalamic astrocytes than in cortical astrocytes as early as P7. As Sparc antagonizes the synaptogenic effect of hevin during glutamatergic synaptogenesis (Kucukdereli et al., 2011), higher Sparc levels may also help maintain a GABAergic synaptic environment in the hypothalamus. This is also consistent with the observation that the growth of astrocyte domain size in subcortical thalamus and hypothalamus is not influenced by the loss of glutamatergic synaptic signaling in VGluT1^–/–^ mice. The higher Sparc levels at P7 also support the notion that the molecular differences between adult cortical and hypothalamic astrocytes are likely to be predetermined in progenitors that are heterogeneously positioned along the VZ during late embryogenesis. Moreover, astrocytes in representative subcortical regions also show a modest growth of their domain sizes and exhibit a significantly smaller domain size compared to that of cortical astrocytes at P30 when astrocytes generally complete postnatal maturation. This likely reflects a difference in astroglial coverage on synapses in these regions, which subsequently affects how astrocytes modulate synaptic signaling.

## Data Availability Statement

No large datasets were generated from this study. All data supporting the findings of this study are available from the corresponding author on reasonable request.

## Ethics Statement

The animal study was reviewed and approved by the Tufts University Institutional Animal Care and Use Committee.

## Author Contributions

TS bred mice and performed the tamoxifen and EdU injections, imaging, cell quantification, immunostaining, and data analysis. AO performed the tamoxifen injections, immunostaining, imaging, quantification, and data analysis. RJ bred mice and performed the immunostaining, imaging, quantification, and data analysis. YM performed the quantification, Ki67 immunostaining, and data analysis. MC performed the immunostaining and mouse breeding. YY designed the overall study, analyzed the data, and wrote the manuscript.

## Conflict of Interest

The authors declare that the research was conducted in the absence of any commercial or financial relationships that could be construed as a potential conflict of interest.
